# Physician’s Leisure Library

**Published:** 1891-12

**Authors:** 


PHYSICIAN'S LEISURE LIBRARY. Electricity: Its Application in Medicine and Surgery. A brief and practical exposition of modern scientific electro-therapeutics. By WELLINGTON ADAMS, M. D., author of Art of Telephony—By Whom Discovered; Evolution of the Electric Railway; Designs and Construction of Dynamo-Electric and Electro-Dynamic Machinery. Lecturer on Electro-Therapeutics, University Medical College, Kansas City; formerly Professor of Diseases of the Ear, Nose and Throat, Medical Department, University of Denver, and Editor *Rocky Mountain Medical Review*. In three volumes. 1891. George S. Davis, Detroit, Mich.

Parts one and two, which have appeared, are devoted to the description of the different batteries, electrodes, and general electrical paraphernalia. The volumes show that the writer is thoroughly familiar with the technique of electrical apparatus. We await, with great hopes, the publication of the third volume, which is to deal with the use of electro-therapy. J. W. P.

